# Anti-Proliferative Effect on Medulloblastoma of Small Metabolites Derived from *Staurosirella pinnata* (Bacillariophyta) Exposed to Different Irradiances

**DOI:** 10.3390/md24060194

**Published:** 2026-05-31

**Authors:** Saverio Savio, Michela Sodini, Matteo Odorisio, Debora Paris, Antonella Guzzon, Marianna Carbone, Maria Letizia Ciavatta, Carlo Rodolfo, Roberta Congestri

**Affiliations:** 1 Department of Biology, University of Rome Tor Vergata, Via della Ricerca Scientifica 1, 00133 Rome, Italy; mhl.sodini@gmail.com (M.S.); matteo.odorisio@gmail.com (M.O.); antonella.guzzon@isprambiente.it (A.G.); carlo.rodolfo@uniroma2.it (C.R.); 2 AlgaRes srl., Via Antonio Silvani 130, 00139 Rome, Italy; 3 Institute of Biomolecular Chemistry (ICB), National Research Council (CNR), Comprensorio Olivetti, Via Campi Flegrei 34, 80078 Pozzuoli, Italy; debora.paris@cnr.it (D.P.); marianna.carbone@cnr.it (M.C.); marialetizia.ciavatta@cnr.it (M.L.C.)

**Keywords:** microalgae biotechnology, diatoms, drug discovery, anti-cancer, *Staurosirella pinnata* bioactivity

## Abstract

An isolate of the diatom *Staurosirella pinnata* is a promising platform for drug discovery due to its ability to produce bioactive metabolites. As previously shown, *S. pinnata* extracts exhibit bioactivities, with hydrophilic fractions showing selective cytotoxicity against human melanoma cells and lipidic fractions promoting thermogenesis in murine white adipocytes. In this work, we focused on the interaction between *S. pinnata* metabolism and light irradiance exposure to evaluate bioactivity targeting medulloblastoma cells. Cultures under standard, control, irradiance (80 µmol photons m^−2^ s^−1^) were exposed in the stationary phase to increased light intensities (200 and 600 µmol photons m^−2^ s^−1^) for 126 h. Growth, photosynthetic performance and metabolic profile were monitored, while the bioactivity of small-molecule fractions was assessed at the end. Exposure to 200 µmol photons m^−2^ s^−1^ significantly enhanced growth (92.6% increase in absorbances compared to the control), whereas 600 µmol photons m^−2^ s^−1^ induced growth inhibition (41.3% decrease in absorbances with respect to the control culture) and impaired photosynthesis. Metabolomic analysis revealed a shift from carbohydrate to lipid metabolism. Bioactivity assays showed that extracts from the highest irradiance exhibited cytotoxic effects on medulloblastoma cells, similar to the 80 µmol photons m^−2^ s^−1^ cultures on DAOY (68% vs. 82% of cell death induction levels, respectively), while intermediate irradiance did not show a significant effect in any of the tested cell lines. The results showed that different light intensities impact *S. pinnata* metabolism, demonstrating effects exploitable for drug discovery and the importance of investigating the impact of cultivation parameters in modulating *S. pinnata* bioactivity potential.

## 1. Introduction

Diatoms constitute a highly diverse group of marine and freshwater microalgae renowned for their ability to synthesise secondary metabolites in response to specific physiological and environmental triggers, such as nutrient limitation, grazing pressure, temperature and light fluctuations that activate intracellular signalling and metabolic pathways, most of which remain to be characterised. The study of diatom cultures in confined, controlled growth systems enables setting of biotic and abiotic factors, thereby circumscribing their effects on the metabolism of selected species. This is particularly important when diatoms are cultivated to produce target bioactive compounds, and this cultivation is taking place in large-scale closed systems or photobioreactors.

In the context of diatom-based drug discovery, our previous investigations [1,2] demonstrated that various bioproducts sequentially extracted from the diatom *Staurosirella pinnata* (Ehrenberg) D.M. Williams & Round exhibit distinct bioactivities against multiple targets. In particular, low-molecular weight hydrophilic fractions showed selective cytotoxic effects against two melanoma cell lines (A375 and CHL-1) while remaining ineffective against normal human keratinocytes (HaCaT) [1]. Furthermore, the bioactivity of a lipidic extract was evaluated in white adipose tissue cells (3T3-L1), revealing a significant modulation of mitochondrial dynamics alongside the enhancement of lipolysis and thermogenesis [2]. Notably, these biological activities were observed when *S. pinnata* was grown under a ‘standard’ light intensity of 80 μmol photons m^−2^ s^−1^ across multiple cultivation runs in laboratory-scale photobioreactors.

The existing literature has reported the effects of light, in terms of intensity, spectral properties and photoperiod, not only as an essential source of energy for diatoms but also in inducing responses at the cell physiological, biochemical and behavioural levels, thus regulating the synthesis of specific compounds with bioactive properties [3,4,5]. For example, light irradiance affected the accumulation of the neurotoxin domoic acid in *Pseudo-nitzschia multiseries*, with maximal synthesis under low irradiance (15 µmol photons m^−2^ s^−1^), while *Skeletonema marinoi*, exposed to 600 µmol photons m^−2^ s^−1^, showed an increased content of ovothiols, with anti-proliferative properties against human cancer cells [6,7]. In addition, the model diatom *Phaeodactylum tricornutum* responded to severe light fluctuation by increasing oxylipin synthesis, which suggests a role for these molecules in signalling [8], while in *Thalassiosira pseudonana*, the exposure to blue light enhanced the production of bioactive lipids such as eicosapentaenoic acid (EPA) [9].

The therapeutic potential of bioactives derived from diatoms is well documented [10,11,12]; thus, a strategy to enhance their biosynthesis in a predictable fashion can be to manipulate key culture parameters as the light regime [13]. In this work, we exposed *S. pinnata* cultures, after the onset of the stationary phase, at higher irradiances, namely 200 and 600 μmol photons m^−2^ s^−1^, for 126 h, to evaluate the effects on its bioactivity. More specifically, we analysed the cytotoxic effect against medulloblastoma cell lines of *S. pinnata* small metabolites (below 3 kDa) isolated by fractionating the diatom hydrophilic extracts. In parallel, we evaluated *S. pinnata* photosynthetic performance under the three light treatments, as well as the impact on cell metabolism by NMR, including the accumulation of stress-related metabolites.

This work tries to fill the gap between *S. pinnata* bioactivity and culture conditions, in particular the effect of light irradiance regulating hydrophilic metabolites that showed anti-proliferative potential against cancer cells. The need for precise control over diatom cultivation protocols when the biomass is intended for biotechnology applications and specifically for drug discovery is thus addressed.

## 2. Results and Discussion

### 2.1. Effect of Light Treatment on S. pinnata Growth

**Figure 1:**
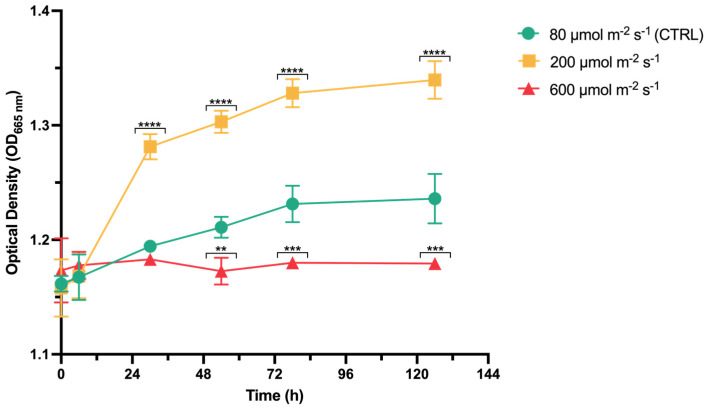
Growth of *S. pinnata* cultures exposed to different irradiances. Cultures were treated with control (80 μmol photons m^−2^ s^−1^, green circles), intermediate (200 μmol photons m^−2^ s^−1^, yellow squares), and high (600 μmol photons m^−2^ s^−1^, red triangles) light irradiances for a total of 126 h. Growth was monitored by measuring the optical density at 665 nm (OD_665_) at the indicated time points. Data represent mean values ± SD (*n* = 3). Significant differences compared to control were assessed by one-way ANOVA followed by Tukey’s post hoc test. Significance was defined as *p*-value < 0.05. **, *p*-value < 0.01; ***, *p*-value < 0.001; ****, *p*-value < 0.0001, compared to control.

The effects of the three irradiance treatments on *S. pinnata* culture were monitored by assessing the cell growth and viability after 6, 30, 54, 72, and 126 h, by means of spectrophotometric measurements of the optical density at the wavelength of 665 nm (OD_665nm_), corresponding to the chlorophyll *a* absorption peak (Figure 1).

Cultures exposed to 200 μmol photons m^−2^ s^−1^ (200I cultures) exhibited a sigmoidal growth pattern similar to control (80 μmol photons m^−2^ s^−1^, 80I cultures). However, the 200I cultures absorbances (OD_665_) were significantly higher, with the steeper increase indicating more active growth and biomass accumulation already after 30 h. Throughout the experiment, the OD_665_ values of 200I cultures remained significantly higher those of both the control and the high-irradiance treatment (600 μmol photons m^−2^ s^−1^, 600I cultures).

At the end of the treatment (126 h), the 200I cultures showed a 92.6% increase in absorbance compared to the control, while the 600I cultures showed markedly limited growth, with OD values plateauing after 30 h and remaining significantly lower those of both 80I and 200I cultures at all time points. In particular, the OD values recorded for the 600I cultures decreased by 41.3% and 69.2% as compared to the 80I and 200I cultures, respectively.

As expected, the highest irradiance treatment limited the diatom growth, likely by inducing photo-inhibitory stress [14,15,16] and reducing biomass production.

### 2.2. Effect of Light Treatment on S. pinnata Photosynthetic Activity

**Figure 2:**
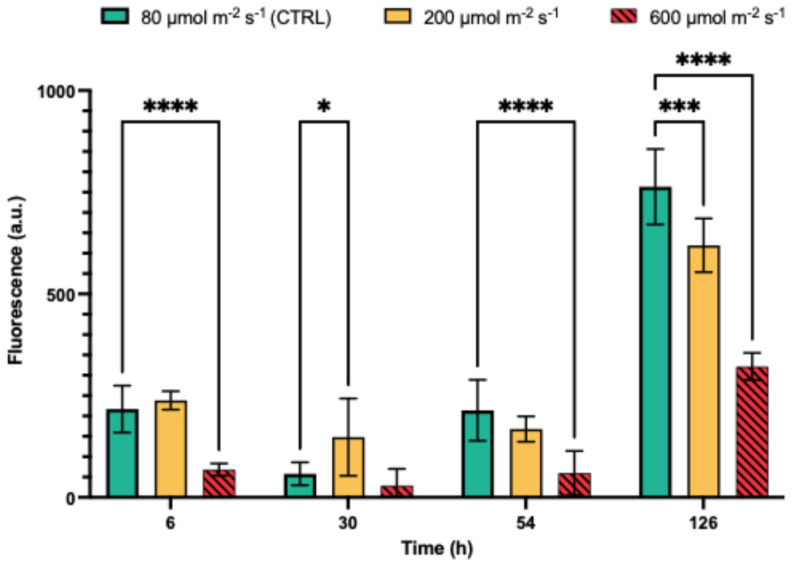
F_0_ values measured in the three cultures treated for 6, 30, 54 and 126 h. Significant differences among treatments were assessed by one-way ANOVA followed by Tukey’s post hoc test. Significance was defined as *p*-value < 0.05 (*), *p*-value < 0.01; ***, *p*-value < 0.001; ****, *p*-value < 0.0001, compared to controls. Data are presented as means ± SD (*n* = 3).

Pulse amplitude modulated measurements were performed at different time points on culture aliquots (5 mL). The minimum dark fluorescence (F_0_), a proxy of photosynthetic active biomass, was measured on 30 min dark-acclimated samples, and the data are summarised in Figure 2.

Overall, the F_0_ values increased over time across all cultures tested, with the steepest rise measured in all samples from 54 to 126 h. In the 80I and 200I cultures, F_0_ increased from ~200 to ~700 a.u., whereas in the 600I cultures the increase occurred between ~70 and ~300 a.u. Pairwise comparisons revealed that the F_0_ values for the 600I cultures were significantly the lowest at all time points, except for the 30 h time point, when no significant differences were recorded. In contrast, the 200I culture values showed a mixed pattern, with no significant differences with respect to the control (80I cultures) after 6 and 54 h; whilst higher F0 were measured at 30 h, and at the end of the experiment (126 h), the values were significantly lower.

**Figure 3:**
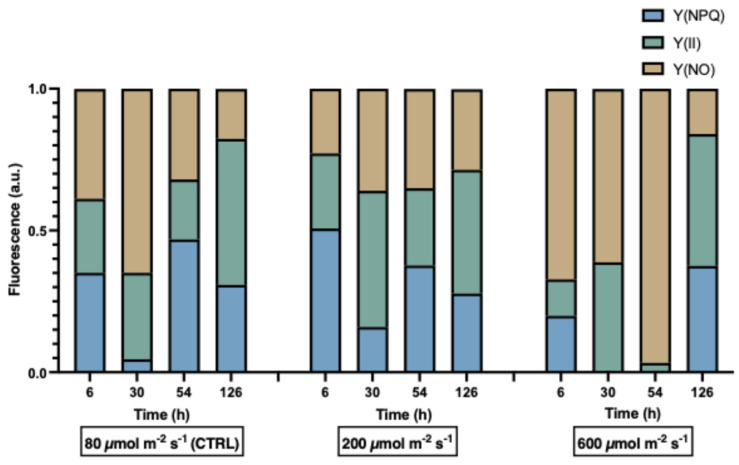
Complementary PSII yields of the steady-state photosynthesis on culture samples after 6, 30, 54, and 126 h. Y(II): efficiency of photosynthetic electron transport in steady-state conditions. Y(NPQ): dissipation of excess energy through photoprotective mechanisms. Y(NO): non-regulated energy loss via heat dissipation.

We furthered the PAM analysis of the different cultures by investigating the complementary PSII yield that describes the energy partitioning in photosystem II. In detail, we measured Y(II), which indicates the efficiency of photosynthetic electron transport in steady-state conditions, Y(NPQ), which estimates the regulated dissipation of excess excitation energy through photoprotective mechanisms, and Y(NO), a proxy of the non-regulated energy loss via heat dissipation and fluorescence emission, thus indicating photodamage severity (Figure 3).

The PSII energy partitioning analysis on 200I cultures, showed increased Y(II) from 0.265 to 0.436 (126 h, *p* = 0.01), while Y(NPQ) declined from 0.507 to 0.278 (*p* = 0.01), indicating an enhanced photochemical efficiency and a reduced need for photoprotective energy dissipation over time. The Y(NO) values remained stable between 0.2 and 0.3 throughout the treatment, without significant temporal variations, revealing minimal photodamage. The 200I data were similar to those of the 80I cultures that showed increased Y(II), from 0.261, measured after 6 h, up to 0.515, recorded after 126 h (*p* = 0.01); while the Y(NPQ) values were maintained around 0.3 throughout the treatment. Finally, Y(NO) declined from 0.387, at the 6 h time point, to 0.176 (126 h), demonstrating improved photosynthetic performance. By contrast, the 600I cultures had a different pattern in PSII energy partitioning. In detail, after a 6 h treatment, the Y(II) and Y(NPQ) values were around 0.1 and 0.2, respectively, while Y(NO) was recorded at 0.7, evidencing severe photoinhibition with the majority of energy lost as heat; however, at 126 h, the values of Y(II) and Y(NPQ) were markedly higher, increasing from 0.1 (6 h) to 0.4 (126 h). This was paralleled by an evident drop in Y(NO) (from 0.7 to 0.1), indicative of a recovery in the photosynthetic performance of the 600I cultures and the photoacclimation capacity of *S. pinnata*. Indeed, by the end of the experiment, the PSII energy partitioning patterns for all treatments converged, reflecting physiological and behavioural adjustment over the 54 to 126 h interval [17].

### 2.3. Metabolic Analysis of S. pinnata Exposed to Different Irradiances

**Figure 4:**
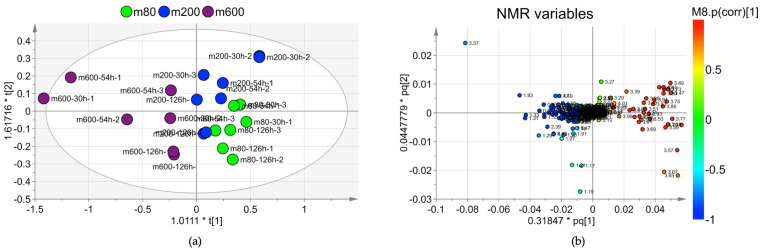
(**a**) Scores plot showing sample projection onto principal components: groups represent *S. pinnata* irradiated with 80 (m80, green dots), 200 (m200, blue dots) and 600 (m600, purple dots) μmol m^−2^ s^−1^ for 30, 54 and 126 h; (**b**) loadings plot reporting the NMR variables (chemical shift) responsible for clustering in the model.

To evaluate metabolic changes in *S. pinnata* cultures exposed to the different irradiances, OPLS-DA was performed on NMR spectra acquired from sample extracts (*n* = 22), as reported in Figure 4. From the scores plot, Figure 4a, we observed spectra projection and discrimination: the first component t[1] accounted for the main differences between metabolites of the 600I cultures (m600) at t[1] negative coordinates *versus* both m80 (metabolites of 80I cultures) and m200 (metabolites of 200I cultures), all placed at positive t[1]. The second component t[2] expressed a minor separation between the control group (m80) and m200. The loadings plot in Figure 4b shows the NMR variables (metabolite chemical shifts) responsible for sample projection and clusters in the model.

By assigning metabolites to the variables expressed in the associated loadings plot in Figure 4b, we found that m600 was characterised by high levels of alanine, lactate, 3-hydroxybutyrate, acetone and short-chain fatty acids, while, on the other side, it had a low content of choline, glycerol and glucose. Following the second component, m80 was mainly characterised by a high content of ethanol, while m200 showed elevated levels of GABA, inosine and uridine.

**Figure 5:**
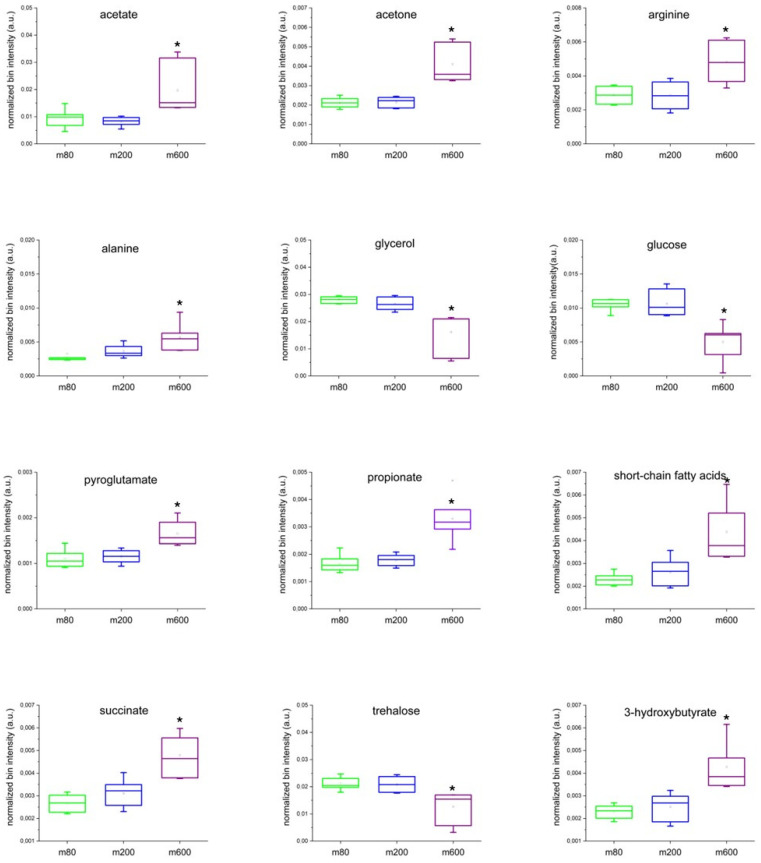
The most modulated metabolites (m80, m200 and m600) in *S. pinnata* irradiance-treated cultures after 6, 30, 54 and 126 h. Significance was defined as *p*-value < 0.05 (*).

To assess a distinct metabolic trend across all cultures, univariate statistical analysis was applied to discriminant metabolites. Bin integrations of statistically significant metabolites are reported as box plots in Figure 5.

The results revealed significant differences in all the analysed metabolites for m600, while comparable levels were observed for m80 and m200, as summarised in Table 1.

**Figure 6:**
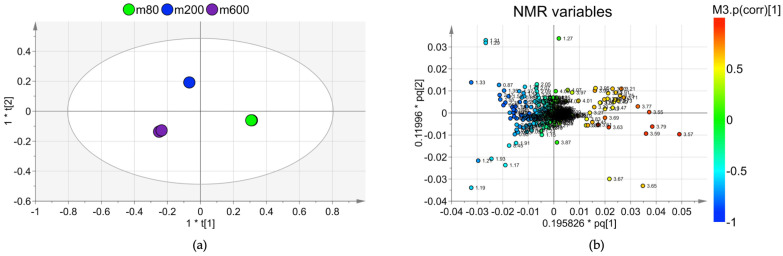
(**a**) Scores plot showing sample projection onto principal components: metabolites of *S. pinnata* cultures exposed to 80 (m80, green dots), 200 (m200, blue dots) and 600 (m600, purple dots) μmol photons m^−2^ s^−1^ for 126 h. (**b**) Loadings plot reporting NMR signals responsible for sample clustering.

To better improve the discrimination between the different cultures, we focused on analysing the metabolic profiles of samples collected only at the end of the experiment (126 h), performing a novel OPLS-DA on all cultures, as shown in Figure 6.

The elaborated statistical model resulted in two predictive and one orthogonal components and parameters R2 = 0.85 and Q2 = 0.58. The scores plot completely discriminated the different cultures’ metabolites. As shown in Figure 6a, the first component t[1] accounts for the main differences between m600 at both t[1] and t[2] negative coordinates; m80 is completely discriminated along the first t[1] component at positive values, while m200, is placed at negative t[1] and positive t[2] values. Indeed, here, the second component t[2] allowed for the differentiation of m600 from m200, resulting in projected negative and positive t[2] coordinates, respectively. Interestingly, by selectively analysing the 126 h samples, we found m200 closer to m600, while m80 had the longest distance along the first principal component.

By examining the associated loadings plot, shown in Figure 6b, depicting NMR signals responsible for the variations leading to sample positioning and clusters in the model, we confirmed the presence of several metabolites characterising m600. However, we identified fewer metabolites contributing to discriminate m80 from m200.

**Figure 7:**
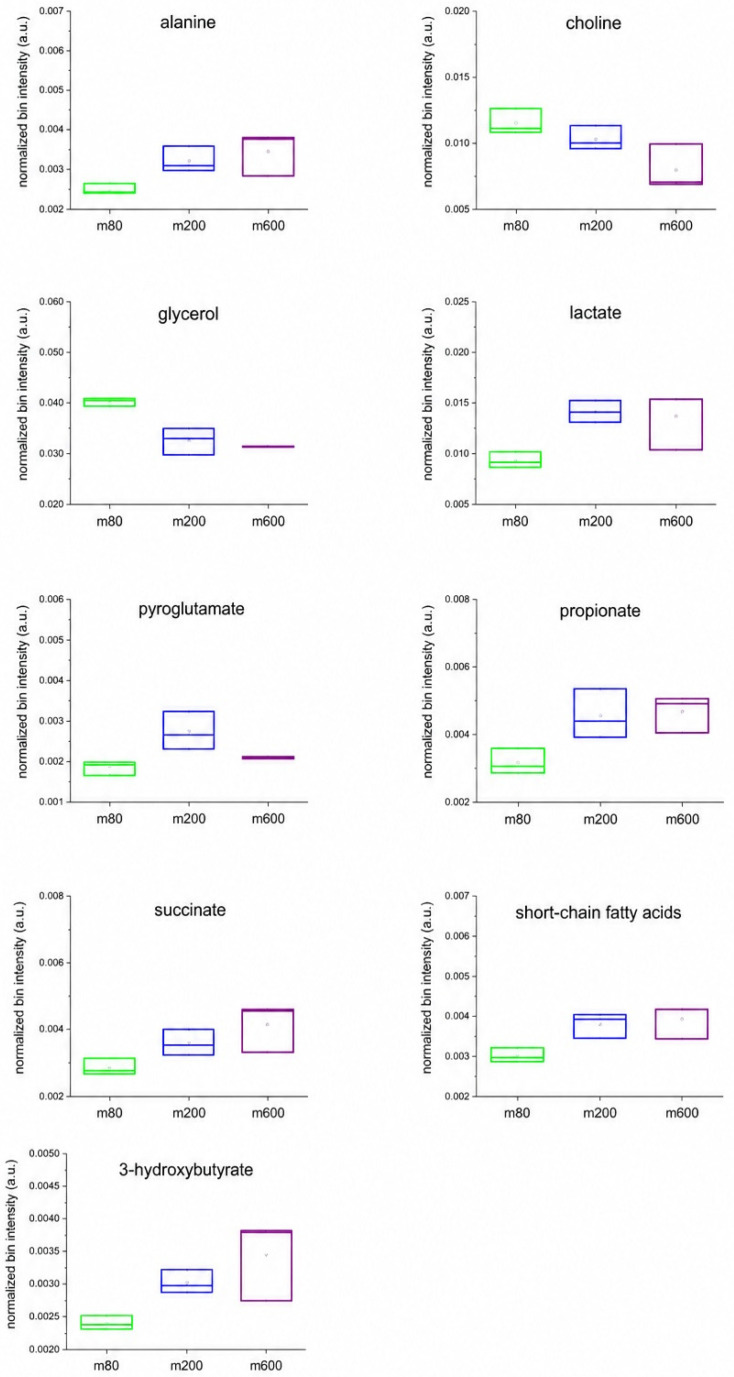
Most modulated metabolites of *S pinnata* cultures exposed to 80 (m80), 200 (m200) and 600 (m600) μmol photons m^−2^ s^−1^ over experimental time.

The associated bin integrations of statistically significant metabolites are presented as a box plot in Figure 7.

In contrast to the data shown in Figure 5, we observed that the 600I and 200I cultures exhibited higher similarity than 80I. This similarity was characterised by comparable levels of alanine, propionate, succinate, short-chain fatty acids, and 3-hydroxybutyrate, coupled with a decrease in the content of choline and glycerol compared to the control (80I cultures).

Overall, the data suggest that the 600I cultures exhibited a significant increase in acetate, acetone, short-chain fatty acids, 3-hydroxybutyrate, pyroglutamate, and arginine concentrations, paralleled by a decrease in glucose, trehalose, and glycerol.

**Figure 8:**
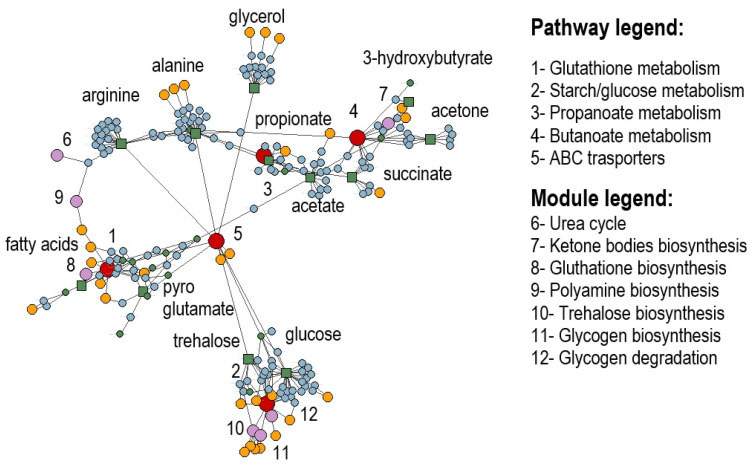
Enrichment analysis of *S. pinnata* metabolites at the different irradiances: m80, m200 and m600. The network is constructed based on the KEGG database results for the model green microalga *Chlamydomonas reinhardtii*. The resulting 211 networks and subnetworks are visualised and filtered using a significance threshold of *p*_adj_ < 0.05. Pathways: red dots; modules: pink dots; enzymes: yellow dots and blue dots; metabolites: green squares and dots.

The enrichment analysis was performed on the most representative metabolites identified in *S. pinnata* cultures across all light intensities (m80, m200, and m600) and time points as reported in Figure 8.

The enrichment analysis data combined with statistically significant metabolite variations (Table 1) revealed a marked metabolic reorganisation in m600 compared to m80 and m200. While no significant differences were observed between the m80 and m200, m600 showed significant shifts in key metabolic pathways, as visualised by the diffusion-based network analysis (Figure 8). The most striking feature of the m600 profile is the shift toward alternative energy sources. In particular, m600 had a significantly lower primary sugar content, evidenced by the marked decrease in glucose and trehalose, which correlates with reduced starch and glucose metabolism (Pathway 2) and trehalose biosynthesis (Module 10). This is combined with lower glycerol, likely diverted to sustain essential gluconeogenic flux. Thus, the system appears to pivot toward ketogenesis and fatty acid derivatives, as indicated by the significant accumulation of acetone and 3-hydroxybutyrate, supported by the activation of ketone body biosynthesis (Module 7). This metabolic shift is further evidenced by the increased levels of acetate, succinate, and propionate that are indicative of enhanced anaplerotic activity within the propanoate and butanoate metabolic pathways (Pathways 3 and 4) in order to maintain the integrity of the tricarboxylic acid cycle. In addition, increased arginine and alanine contents suggest a more active urea cycle (Module 6) in regulating nitrogen homeostasis or addressing increased protein turnover. Furthermore, higher pyroglutamate levels highlight an active involvement of glutathione metabolism (Pathway 1) and biosynthesis (Module 8), suggesting an adaptive mechanism aimed at mitigating oxidative stress in m600. These shifts indicate that m600 represents a distinct phenotype characterised by a transition from glycolytic reliance to ketogenesis and organic acid accumulation. The integration of statistical trends with diffusion-based pathway mapping confirms that these changes are not isolated events but part of a coordinated systemic response involving energy production, nitrogen balancing and redox homeostasis.

This metabolic profile suggests a shift from carbohydrate to lipid metabolism, consistent with stress-response mechanisms observed in other microalgae, under adverse light conditions [18]. In particular, the accumulation of 3-hydroxybutyrate and pyroglutamate aligns with documented stress-response pathways in diatoms and other microalgae, where these compounds contribute to oxidative stress mitigation [19,20,21]. Similar metabolic reprogramming has been reported for *Skeletonema marinoi* and *Chaetoceros tenuissimus,* under nitrogen limitation, where stress metabolites accumulate during stationary growth phases [22,23]. We hypothesise that the stress-induced metabolic flux redirection observed here represents an adaptive mechanism to maintain cellular homeostasis under high-irradiance conditions, as previously reported by Osundeko and colleagues [24].

### 2.4. Bioactivity of S. pinnata Small Metabolite Fractions on Medulloblastoma Cells

**Figure 9:**
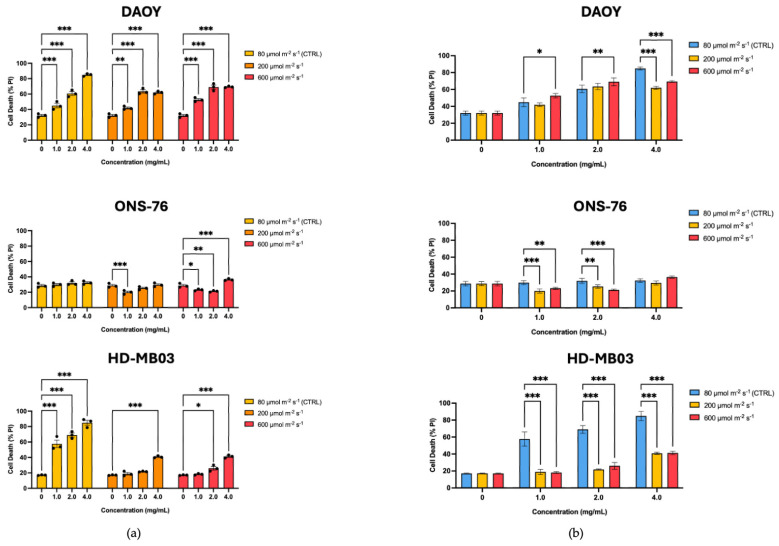
(**a**) Cell death induction after administration of extract fraction containing metabolites with a molecular mass below 3 kDa on DAOY, ONS-76, and HDMB03 medulloblastoma cell lines. (**b**) Comparative responses of the three medulloblastoma cell lines to the different irradiance-derived extracts across all tested concentrations. For the experiment, cell lines were incubated for 24 h with the indicated amount of extract, and cell death was quantified as the percentage of hypodiploid events. Significance was defined as *p*-value < 0.05 (*), *p*-value < 0.01 (**), and *p*-value < 0.001 (***), compared to the control. Data are presented as means ± SD (*n* = 3).

To assess the bioactive potential of the fractions composed of small metabolites (below 3 kDa) obtained from *S. pinnata* cultures exposed to the different irradiances, we performed dose–response assays on three medulloblastoma cell lines: DAOY, ONS-76, and HD-MB03 (Figure 9).

As shown in Figure 9a, the DAOY cell line exhibited a pronounced dose-dependent sensitivity towards all the tested extracts, with the 80I-derived showing the highest efficacy (82% cell death at 4.0 mg/mL). In contrast, administration of the 200I- and 600I-derived fractions resulted in a significantly lower cytotoxicity, with cell death rates of approximately 60% at 4.0 mg/mL (Figure 9b). These findings suggest that lower irradiance conditions favour the production/accumulation of bioactive molecules with higher cytotoxicity potential. On the other hand, administration of the same extracts to the ONS-76 cell line resulted in a markedly different response, with the cytotoxic effect considerably reduced. Indeed, only the 600I-derived fraction exhibited a slight cell death induction potential (38% at 4.0 mg/mL), while the 80I- and 200I-derived fractions did not exert any significant effect. Finally, the HD-MB03 cell line displayed an intermediate sensitivity to the extracts, with a response profile falling between that of DAOY and ONS-76. The 80I cultures fraction induced a significant dose-dependent increase in cell death, resembling the pattern observed in DAOY cells. However, the 200I- and 600I-derived fractions showed a weaker but similar cytotoxic effect, resulting in cell mortality rates below 40% at the highest tested concentration.

As reported in Figure 9b, comparative analyses of the three medulloblastoma cell lines’ responses confirmed the efficacy of the extracts towards the DAOY cell line, with a clear dose-dependent trend, independent of the irradiance value. On the other hand, the HD-MB03 cell line displayed a strong dose-dependent effect only for the 80I-derived extract, while the 200I- and 600I-derived ones showed a significantly reduced impact. In this case, the ONS-76 cell line not only showed a completely different behaviour in terms of cytotoxicity, but also it looks like the 200I- and 600I-derived extracts exert a pro-survival effect at intermediate (1.0 and 2.0 mg/mL) concentrations.

Taken together, these results showed that standard (80I) irradiance conditions allow for the production/accumulation of bioactive small molecules able to induce significant cell death levels in DAOY and HD-MB03 MB cell lines, and higher irradiances result in a slight reduction in this bioactive potential for the DAOY cell lines and in a more significant one for HD-MB03. As for the ONS-76, this cell line looks to be only slightly responsive to the cytotoxic activity of the tested extracts, also showing some kind of beneficial effect. The observed differences might be related to the different genetics, biology, metabolism, aggressiveness, and therapeutic responses that characterise the different MB sub-groups. Indeed, it is known that the Sonic Hedgehog (SHH), comprising DAOY and ONS-76, and the Group 3 (Gr.3), comprising HD-MB03, sub-groups are very different, especially in terms of aggressiveness and response to therapy. We could hypothesise that the different composition of the high-irradiance extracts has less impact on HD-MB03, because of their stem-like nature and their reliance more on glycolysis and the TCA cycle, rather than oxidative phosphorylation, for energy production. On the other hand, even if the ONS-76 cell line belongs to the SHH subgroup, they differ from the DAOY by bearing wild-type p53 and being more differentiated and less aggressive. In this case, we could hypothesise that both these aspects, by relying on different pathways activation, could be responsible for the observed behaviour.

To fully address this point, we need to perform a more detailed analysis of the cell-specific response by assessing, for instance, mitochondrial functionality, cellular metabolism, and pro-survival vs. pro-death pathways’ activation [10,11,12]. The observed modulation of metabolites in response to the varied light environments underscores the dynamic nature of *S. pinnata* physiology and its potential for ‘customising’ the production of target bioactive compounds.

## 3. Materials and Methods

### 3.1. Staurosirella pinnata Cultures and Light Treatment

A strain of the araphid chain-forming diatom species *Staurosirella pinnata* (Ehrenberg) D.M. Williams & Round (strain VRUC 290 of Tor Vergata Rome University Collection) was isolated from biofilms colonising sediments of a Mediterranean coastal lagoon (Cabras, Sardinia, Italy) [25]. For the experiments, an inoculum of 6 L of *S. pinnata* was prepared using Diatom Medium (DM) and acclimated to 80 μmol photons m^−2^ s^−1^ for two weeks. Then, cultures (*n* = 3 for each light condition tested) were irradiated for 126 h, using 200 and 600 μmol photons m^−2^ s^−1^ using a 12:12 h light/dark cycle, with the 80 μmol photons m^−2^ s^−1^ (Lamp T8 G13 L 18 W/77, OSRAM^®^ FLUORA Premstaetten, Premstaetten, Austria) illuminated culture serving as control. The culture growth was monitored after 6, 30, 54 and 126 h, measuring the optical density (OD) of 1 mL samples (*n* = 3) at λ = 665 nm and 730 nm (Onda UV-30 Scan spectrophotometer, ^®^Giorgio Bormac S.r.l., Carpi, Italy). To evaluate the possible presence of contaminants, culture samples were also analysed by light microscopy (Zeiss Axioskop, 40×, GmbH, Jena, Germany), over the whole growth period.

At the end of the irradiance treatments (126 h), the biomass was harvested by settling and centrifuging (2.500× *g*, 10 min) and then freeze-dried (Edwards SO4) and stored at −40 °C.

### 3.2. Culture Photosynthetic Activity Under Light Treatment

The photosynthetic activity of *S. pinnata* cultures under the three different irradiances was assessed using a Miniaturised Pulse Amplitude Modulated (Mini-PAM) fluorometer, coupled to WinControl Software 3.40 (Walz GmbH, Effeltrich, Germany) for computer control and data analysis. Fluorescence was excited by pulses (3 µs width) of measuring red light from a light-emitting diode (LED) with the peak excitation band at 650 nm. Fluorescence measurements were performed on light acclimated samples to assess the effective quantum yield of the charge separation Y(II) in photosystem II (PSII), and the measurements were performed on light-acclimated samples. Three 2 mL aliquots of *S. pinnata* (*n* = 3 for each light condition tested) were sampled after 6, 30, 54 and 126 h of irradiance treatment from each culture replicate and positioned into multi-well plates, to obtain separate spots for measurements. A Mini-PAM fibreoptic was kept at 10 mm above the sample surface using a holder to obtain a homogeneous field of light [26,27]. The Y(II) of the light acclimated cultures was calculated according to (F_m_′ − F)/F_m_′, where F is the steady-state fluorescence of the light-acclimated sample, and F_m_′ is the maximum light-acclimated fluorescence after the application of a single saturating light pulse (0.8 s, 3000 µmol photon m^−2^ s^−1^) [26,27].

After Y(II) measurement, the samples were dark acclimated for 30 min to determine the minimum fluorescence level F_0_ and investigate non-photochemical quenching (NPQ) related parameters, namely Y(NPQ), which is the amount of energy dispersed as non-photochemical processes, and Y(NO), which is the amount of energy lost by heat dissipation. F_0_ was then determined with a measuring light (peak at 650 nm). A 0.8 s flash of saturating light was given to determine the maximum fluorescence, F_m_. The fluorescence parameters were calculated according to Oxborough and Baker [28].

### 3.3. Metabolomic Analysis

#### 3.3.1. Biomass Extraction for NMR Analysis

To evaluate the metabolite contents of the different cultures, aliquots of 15 mL samples were collected from cultures subjected to 80, 200 and 600 µmol photon m^−2^ s^−1^ after 30, 54, and 126 h (*n* = 3 for each irradiance condition and time-point). The samples were then lyophilised, homogenised in liquid nitrogen, and stored at −80 °C. Subsequently, to obtain polar and non-polar fractions, the lyophilised biomass was extracted using a solution of CH_3_OH, CHCl_3_ and ultrapure H_2_O (2:1:1 *v*/*v*/*v*), at 4 °C for 8 h.

#### 3.3.2. NMR Spectra Acquisition of Polar Fractions

Metabolite analysis was performed on the polar phases by resuspending the fractions in 630 µL of phosphate buffer saline (PBS, pH 7.4) and adding 70 µL of 2H_2_O solution (containing 1 mM sodium 3-trimethylsilyl [2,2,3,3-2H4] propionate (TSP) as a chemical shift reference for 1H spectra) to provide a field frequency lock, reaching 700 µL of total volume. Samples were loaded into the autosampler, and NMR spectra were acquired on a Bruker Avance III–600 MHz spectrometer (BrukerBioSpin GmbH, Rheinstetten, Germany), equipped with a TCI CryoProbe fitted with a gradient along the *Z*-axis, at a probe temperature of 27 °C. In particular, standard 1D proton spectra and 2D experiments (clean total-correlation spectroscopy TOCSY and heteronuclear single quantum coherence HSQC) were acquired, providing mono-dimensional (1D) metabolic profiles and homonuclear and heteronuclear spectra for metabolite identification. Metabolite assignment was achieved by comparing the signal chemical shifts with the literature and online databases. All 1D spectra were processed, and the data were automatically reduced in bins, arranged as a data matrix, and imported for multivariate statistical analysis with projection methods.

#### 3.3.3. Metabolite Data Processing and Statistics

All acquired spectra were automatically reduced to 410 integral segments of 0.02 ppm each between the 0.60 and 8.80 ppm spectral region, excluding the water resonance (4.46–5.25 ppm) using the AMIX 3.9.15 software package (Bruker Biospin GmbH, Rheinstetten, Germany). After reducing the NMR data, bins were normalised to the total spectrum area. The obtained data format, expressed by a matrix (X matrix), was then imported into the SIMCA-P+14 package (Umetrics, Umeå, Sweden), where multivariate statistical analysis was performed to discriminate the culture response to the different irradiances provided, according to the corresponding NMR profiles. In particular, unsupervised principal component analysis (PCA) was applied to assess class homogeneity, uncover data trends and detect outliers. Then, Orthogonal Partial Least Squares Discriminant Analysis (OPLS-DA) was used to visualise class separation and the spectral variables influencing samples distribution, according to the alteration of the metabolic profiles under the irradiances used for 30, 54 and 126 h. Data visualisation was achieved through scores and loadings plots, highlighting specific compounds as putative markers useful for classification. OPLS-DA models were evaluated through R2 (goodness of fit) and Q2 quality parameters (power in goodness of prediction) and validated by internal iterative cross-validation with a 7-round permutation test response (800 repeats) and CV-ANOVA (ANOVA testing of Cross-Validated predictive residuals). Selected and isolated signals and bins with |pcorr| ≥ 0.7, VIP > 1 (Variable Importance in the Projection) were considered for univariate statistical analysis elaborated with the OriginPro 9.1 software package (OriginLab Corporation, Northampton, MA, USA) and R software [R core team (https://www.r-project.org/ accessed on 23 May 2025)]. Statistical significance for selected and isolated metabolites was determined by parametric ANOVA with Tukey correction or the non-parametric Kruskal–Wallis test with Dunn–Bonferroni post hoc correction, according to the results of the normality test performed on data to evaluate each distribution (Shapiro–Wilk test). To account for multiple comparisons in the metabolomics analysis, raw *p*-values were further adjusted using the Benjamini–Hochberg False Discovery Rate (FDR) procedure. Adjusted *p*-values (*p*_adj_) < 0.05 were considered statistically significant.

#### 3.3.4. Enrichment Analysis

We performed enrichment analysis on selected and more representative metabolites found in *S. pinnata* cultures irradiated at 80, 200 and 600 μmol m^−2^ s^−1^ for 30, 54 and 126 h using the ‘diffusion’ method computed by the FELLA package in R. Starting from the set of altered compounds in diatom profiles acquired in different conditions, this analysis suggests affected reactions, enzymes, modules and pathways using label propagation in a knowledge model network based on *Chlamydomonas reinhardtii* green microalga database in KEGG. The resulting network and subnetwork are visualised and exported in a related plot and table with a threshold of *p* < 0.05.

### 3.4. Bioactivity Assays on Human Medulloblastoma Cell Lines

#### 3.4.1. Biomass Extraction for Bioactivity Assays

The biomass of cultures treated with the three irradiances was harvested at the end of the experiment (126 h) by settling and centrifugation (4000× *g,* 10 min) and then lyophilised and extracted using an aqueous-methanol solution (20% *v*/*v*) as reported by Savio and collaborators [1,2]. The hydrophilic fractions were then processed by using Amicon^®^ Ultra 3 kDa centrifugal filters (Merck, Darmstadt, Germany). Fractions containing molecules with a molecular mass below 3 kDa (hereafter referred to as X < 3) were dried by using a rotary vacuum (Buchi^®^ Rotavapor, Flawil, Switzerland) at room temperature, stored at −20° C, and lyophilised.

#### 3.4.2. Medulloblastoma Cell Lines

*S. pinnata* X < 3 hydrophilic fractions’ bioactivity was evaluated on DAOY (HTB-186, ATCC culture collection), ONS-76 (ABC-TC0875, ACCEGEN Biotechnology), and HD-MB03 (ACC740, DMSZ culture collection) medulloblastoma cell lines. The DAOY cell line was cultured in Modified Eagle Medium (MEM, Euroclone, Pero, Italy) supplemented with stable glutamine (2 mM), while the ONS-76 and HD-MB03 cell lines were maintained in Roswell Park Memorial Institute (RPMI) 1640 medium (RPMI 1640, Euroclone, Pero, Italy), supplemented with stable glutamine (2 mM). Culture media were further supplemented with 100 IU/mL penicillin, 100 µg/mL streptomycin (Euroclone, Pero, Italy), and 10% (*v*/*v*) heat-inactivated foetal bovine serum (FBS, Thermo Scientific, Waltham, MA, USA). Cells were incubated at 37 °C in a humidified atmosphere with 5% (*v*/*v*) CO_2_.

#### 3.4.3. Bioactivity Assay of the Low-Molecular-Weight Fractions on Medulloblastoma Cell Lines

For bioactivity assays, 5 × 10^4^ cells, seeded in 12-well plates, were administered with 0.5, 1.0, 2.0, and 4.0 mg/mL of the X < 3 extract (*n* = 3 for each dose tested). Cellular responses were evaluated 24 h later by flow cytometry assessment of cell death and cell cycle, upon propidium iodide (50 µg/mL PI, Sigma-Aldrich, Darmstadt, Germany), as previously described [29]. Flow cytometric analyses were performed by using a BD Accuri™ C6 Plus Flow Cytometer (Becton, Dickinson and Company, Franklin Lakes, NJ, USA), with excitation at 488 nm and an emission filter for PI detection (610–620 nm). A minimum of 10,000 events per sample was acquired. Statistical analysis was performed by means of two-way ANOVA using GraphPad Prism 10.0 (GraphPad Software, Boston, MA, USA).

### 3.5. Statistical Analysis

Statistical analyses were performed using one-way or two-way ANOVA (in case of multiple samples) or *t*-test (for two samples), as provided by Prism software (v10.1.1), in combination with the Tukey Honestly Significant Difference (HSD) test, for multiple post hoc testing or Wilcoxon pairwise test (for repeated paired samples), or Mann–Whitney test, for repeated unpaired samples. Correlations among photosynthetic parameters of light-acclimated samples and among fluorescence parameters of dark-acclimated samples were assessed by applying Pearson’s r or Spearman’s rho-statistic. Significance was defined as *p* < 0.05 (*), *p* < 0.01 (**), *p* < 0.001 (***) and *p* < 0.0001 (****).

## 4. Conclusions

The integration of growth, photosynthesis, metabolomic, and bioactivity data reveals complex relationships that are relevant for the potential biotechnological exploitation of *Staurosirella pinnata*.

Indeed, from a biotechnological perspective, these findings contribute to understanding the light-dependent regulation of metabolites in diatoms. *S. pinnata* demonstrates that irradiance represents a crucial parameter for influencing its bioactive potential. The capacity to maintain bioactivity under distinct metabolic states may offer advantages for scaled cultivation systems, potentially addressing reproducibility challenges in diatom-based bioactive compound production [13,30,31,32].

Further research should focus on identifying and characterising the specific compounds responsible for the observed anti-proliferative effects, determining their mechanisms of action, and evaluating cultivation parameters beyond irradiance that may influence the production of *S. pinnata* bioactives. Finally, molecular-level investigation of the biosynthetic pathways involved in bioactive metabolite production under different irradiance conditions would provide a mechanistic understanding applicable to cultivation optimisation for pharmaceutical applications.

## Figures and Tables

**Table 1:** FDR-adjusted *p*-values for metabolites in *S. pinnata* cultures. Comparisons are shown between cultures exposed to high irradiance (m600: 600 μmol photons m^−2^ s^−1^) and low-to-moderate light (m80 and m200: 80 and 200 μmol photons m^−2^ s^−1^). *p*_adj_ values were calculated using the Benjamini–Hochberg False Discovery Rate (FDR) procedure. No significant differences (n.s., *p*_adj_ > 0.05) were observed between m80 and m200. Arrows (↑/↓) indicate the metabolic trend in response to highlight stress (m600) compared to the other conditions.

Metabolite	m80 vs. m200 (*p*_adj_)	m600 vs. m80 (*p*_adj_)	m600 vs. m200 (*p*_adj_)	Trend (vs. m600)
Acetate	n.s.	8.00 × 10^−3^	2.18 × 10^−3^	↑
Acetone	n.s.	4.50 × 10^−5^	8.28 × 10^−5^	↑
Arginine	n.s.	2.18 × 10^−3^	1.33 × 10^−3^	↑
Alanine	n.s.	6.13 × 10^−4^	1.00 × 10^−2^	↑
Pyroglutamate	n.s.	3.45 × 10^−4^	7.37 × 10^−4^	↑
Propionate	n.s.	4.50 × 10^−5^	1.38 × 10^−4^	↑
Short-chain fatty acids	n.s.	3.45 × 10^−4^	1.33 × 10^−3^	↑
Succinate	n.s.	4.50 × 10^−5^	3.36 × 10^−4^	↑
3-Hydroxybutyrate	n.s.	1.85 × 10^−4^	4.20 × 10^−4^	↑
Glycerol	n.s.	4.50 × 10^−5^	1.56 × 10^−4^	↓
Glucose	n.s.	1.92 × 10^−4^	1.56 × 10^−4^	↓
Trehalose	n.s.	2.18 × 10^−3^	2.18 × 10^−3^	↓

## Data Availability

Data are contained within the article.
